# Multiple-locus variable number of tandem repeats analysis of *Salmonella enterica* serotype paratyphi A from Yuxi and comparison with isolates from the Chinese Medical Culture Collection Center

**DOI:** 10.3892/mmr.2014.2187

**Published:** 2014-04-24

**Authors:** YINGBO YAO, XIAOYAN CUI, QINGSHAN CHEN, XINRONG HUANG, BRADLEY ELMORE, QING PAN, SHUKUN WANG, JIE LIU

**Affiliations:** 1 Center for Disease Control and Prevention of Yuxi City, Yuxi, Yunnan 653100, USA; 2 Translational Center for Stem Cell Research, Tongji Hospital, Stem Cell Research Center, Tongji University School of Medicine, Shanghai 200065, USA; 3 Department of Epidemiology, School of Medicine, Jinan University, Guangzhou, Guangdong 510632, USA; 4 Huanggang Maternal and Child Care Service Centre, Huanggang, Hubei 438100, USA; 5 College of Pharmacy, University of New Mexico Health Sciences Center, Albuquerque, NM 87131, USA; 6 Department of Medical Microbiology, Yongzhou Vocational and Technical College, Yongzhou, Hunan 425006, P.R. China

**Keywords:** *Salmonella enterica* serotype paratyphi A, multiple-locus variable number of tandem repeat analysis

## Abstract

The aim of the present study was to genotype *Salmonella enterica* serotype paratyphi A (SPA) isolated from Yuxi, China, in a multiple-locus variable number of tandem repeats (VNTRs) analysis (MLVA) and to compare them with isolates from the Chinese Medical Culture Collection Center (CMCC). Potential VNTRs were screened from the genomes of ATCC9150 and AKU_12601 using the Tandem Repeats Finder program. Nine VNTRs were established for MLVA typing of 195 SPA isolates from Yuxi and 20 isolates from CMCC. The dendogram for MLVA profiles and minimum spanning tree (MST) were drawn using the categorical coefficient calculated by BioNumerics software. A total of 23 MLVA types were identified in 215 SPA isolates and were grouped into six distinct cluster groups A, B, C, D, E and F. A total of 195 Yuxi SPA isolates were exclusively grouped into cluster C with nine MLVA genotypes. A total of 20 CMCC isolates were grouped in clusters A B, D, E and F with the other 14 MLVA types. The MLVA with nine VNTR loci, which was exploited in the present study, represents a successful strategy for genotyping SPA. Furthermore, the 195 Yuxi isolates appear to be closely related to each other and distinct from the 20 CMCC strains.

## Introduction

Infectious diseases caused by a variety of *Salmonella enterica* serotypes are widespread worldwide, representing a severe public health concern (1). Infection with *Salmonella enterica* serotype paratyphi A (SPA) is an emerging global public health problem due to the increase in enteric fever cases caused by SPA and the lack of protective vaccines (2–4). In Southeast and Southwest China, the infection rate of SPA has increased in the past several decades with the development of tourism, where >80% of the enteric fever outbreaks are caused by SPA (5). In recent years, Yuxi City of Yunnan Province has become one of the most severely endemic areas of SPA in China (6).

Subtyping and tracking individual strains involved in SPA outbreak or sporadic cases are important for the control and prevention of SPA transmission in Yuxi. The technique of pulsed-field gel electrophoresis (PFGE) is currently the standard method for molecular typing and epidemic surveillance of *Salmonella* spp., including SPA (7,8). However, PFGE is not a routine method for SPA surveillance due to the expense of the equipment and the requirement of highly trained technicians (9). Multi-locus variable number tandem repeat (VNTR) analysis (MLVA), a genotyping method based on polymerase chain reaction (PCR) and sequencing, which distinguishes tandem sequence repeats that vary in copy numbers (10,11), may be practical for subtyping SPA due to the simple operation, low cost, high-speed and weak laboratory-dependence (12). Furthermore, MLVA genotyping is becoming an important DNA-based typing tool for investigating strains that are related or unrelated to outbreaks (13).

Although one study has previously investigated the use of MLVA for subtyping SPA, the information of VNTRs for MLVA of SPA in this investigation is limited as the VNTRs were examined from the genomes of one strain of SPA (ATCC9150) and two strains of *S. enterica* serovar Typhi (S. Typhi; CT18 and Ty2) (14). Although the genomes of S. Typhi and SPA are closely related (15), their tandem repeats (TRs) are different. The present study searched for TR loci from two SPA genomes, ATCC9150 (NC_006511) and AKU_12601 (NC_011147), and determined nine VNTR loci for MLVA typing of SPA. We aimed to identify the type of epidemic clone in Yuxi and whether the Yuxi SPA isolates were phylogenetically distant from the 20 strains of SPA isolates collected by the Chinese Medical Culture Collection Center (CMCC).

## Materials and method

### Strains and extraction of bacterial genomic DNA

A total of 215 strains of SPA, including 195 Yuxi isolates and 20 CMCC strains were used in the present study. Among the 20 CMCC strains, one strain was ATCC9150 while the other 19 were collected from various research organizations with limited background information and stored by CMCC (Table I). Among the 195 Yuxi isolates, 48 were separated from the patients of the SPA outbreak in 2007 while the others were isolated from sporadic cases between 2005 and 2009.

Genomic DNA of SPA was extracted as previously described (16,17). Briefly, the bacteria were streaked on brain heart infusion agar (BHIA) plates and grown at 37°C overnight in 5% CO_2_ incubator. A loop of typical colonies was removed from the BHIA plates and boiled for 10 min in 200 μl Tris-EDTA buffer (10 mM Tris-Cl and 1 mM EDTA, pH 8.0). The supernatant was obtained by centrifugation at 8,000 × g for 10 min and used directly for PCR (18).

### Identification of VNTRs

**Figure 1:**
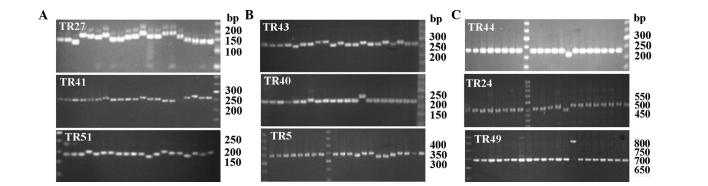
The polymorphisms of nine VNTR loci TR27, TR51, TR41, TR43, TR5, TR40, TR44, TR24 and TR49 analyzed by agarose gel electrophoresis, capillary electrophoresis and sequencing for PCR products amplified from 20 CMCC strains and one Yuxi isolate YN07044. (A) The agarose gel electrophoresis for PCR products of nine VNTR loci. Lanes: left to right, ATCC9150, 50078, 50001, 50502, 50506, 50501, 50505, 50154, 50434, 50101, 50507, 50509,50672, 50674, 50084, 50433, 50508, 50701, 50002, 50504, YN07044. DNA Marker, 50bp DNA Ladder Marker. (B) The representative electropherogram from pooled capillary electrophoresis runs of FAM-labeled or HEX-labeled primers of nine VNTRs. The PCR products for TR27, TR41, TR51, TR43, TR40 and TR5 were amplified from YN07044, demonstrating 143, 259, 194, 276, 217 and 338 bp, respectively. The PCR products for TR44 and TR24 were amplified from 50504 with 254 and 527 bp respectively. (C) The precise numbers of TR40 in three representative sequences amplified from ATCC9150 (3), 50154 (4), and 50674 (6) were analyzed by sequencing. CMCC, Chinese Medical culture Collection Center; SPA, *Salmonella enterica* serotype paratyphi A; VNTR, variable number of tandem repeats.

Potential TRs were first exploited from the genomes of ATCC9150 and AKU_12601 using the Tandem Repeats Finder (TRF) program (19,20) and the http://tandem.bu.edu/trf/trf.htlm website (21). The candidates were scored as match(+2), mismatch(−3) and indel(−5) for pattern alignment (22). The potential TRs were selected by alignment scores ≥80, or homology of repeat locus ≥85%. A total of 51 TRs (TR1-51) were screened from the genomes of ATCC9150 and AKU_12601 (data not shown). Primers flanking >51 TRs were designed using the Primer 5.0 software (Premier Biosoft International, Palo Alto, CA, USA) and synthesized by Sangon Company (Shanghai, China). The polymorphism of PCR fragments amplified with primers of 51 TRs was analyzed by agarose electrophoresis and nine VNTR loci (TR27, TR51, TR41, TR43, TR5, TR40, TR44, TR24 and TR49) were verified to be polymorphic (Fig. 1). The nine VNTRS of 19 CMCC strains except ATCC9150 were sequenced. The repeat numbers for each locus corresponding to 20 CMCC and AKU_12601 are summarized in Table II.

### PCR and agarose electrophoresis analysis

All selected loci were amplified from the genomic DNA of the 20 CMCC SPA strains by PCR as described previously (23). Briefly, 1 μl bacterial lysate was amplified by a thermal cycler PTC-200 DNA Engine (MJ Search Partners, Inc., Lake Forest, IL, USA) in a 25 μl final reaction volume containing 0.1 μmol/ml dNTPs, 0.2 μmol/ml primers, 0.5 U *Taq* DNA polymerase (Takara Bio, Inc., Shiga, Japan) under the following conditions: 10 min at 95°C, followed by 30 cycles of three temperatures (15 sec at 95°C, 1 min at 55~60°C, 1 min at 72°C) and then 10 min at 72°C. A total of 5 μl of the PCR products were separated in 1.5% agarose gels in 1X TAE buffer (AppliChem Inc., St. Louis, MO, USA) at a voltage of 6 V/cm for ~3 h. The gels were stained in ethidium bromide for visualization under UV light and were photographed on a Gel Doc 2000 system (Bio-Rad, Hercules, CA, USA). The 50 bp (base pair) DNA Ladder Marker (Takara Bio, Inc.) was loaded in all of the gels to facilitate determining the size of the DNA fragments. To ensure the accuracy of agarose electrophoresis and to compare the results between multiple gels, the PCR products of ATCC9150 in each locus were obtained as a positive control. The TRs were identified to be polymorphic if large differences between their PCR fragments in the agarose gel electrophoresis were observed. The PCR products were purified with the QIAquick PCR Purification kit (Qiagen, Hilden, Germany) following the manufacturer’s instructions.

### MLVA typing and data analysis

In order to confirm that any length polymorphism of fragment was due to variations in the VNTR copy number (24), the purified PCR products amplified from 195 Yuxi isolate and 20 CMCC strains were sequenced by the Sangon Company. The numbers of repeats in each allele were analyzed by BioNumerics version 6.0 (Applied Maths, Austin, TX, USA) (25), and the numerical profile for each locus was created according to the copies of VNTR (14,26). The dendogram for MLVA profiles was drawn using the categorical coefficient and the alignment of unweighted pair group method using arithmetic averages (27). A minimum spanning tree (MST) was constructed using the categorical coefficient (10,23,28). The priority rule for constructing MST was set so that the genotypes that had the highest number of single-locus variants would be linked first (23).

## Results

### MLVA genotyping and phylogenetic analysis for 215 SPA isolates

**Figure 2:**
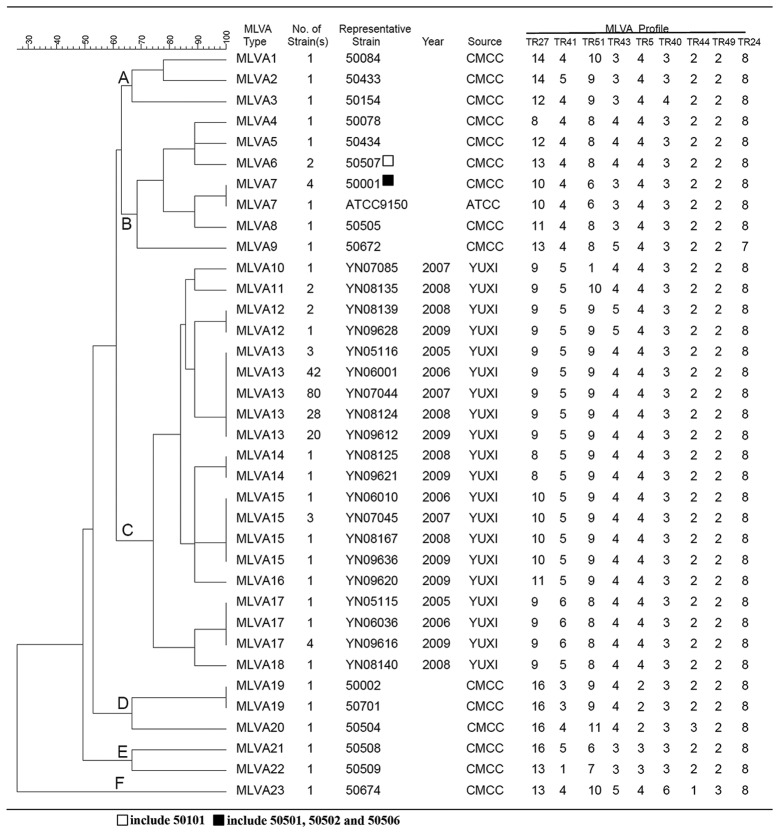
MLVA type distribution of 215 SPA isolates using categorical coefficient and unweighted pair group method using arithmetic average. The 215 SPA isolates, including 195 Yuxi isolates and 20 CMCC strains were subtyped into 23 MLVA types (MLVA1~23) and grouped into six distinct clusters (A, B, C, D, E and F). All of the 195 Yuxi isolates were grouped in the C cluster with nine MLVA genotypes (MLVA10~18). The 20 CMCC strains were grouped into A, B, D, E and F clusters with 14 MLVA types (MLVA 1-9 and MLVA 19-23). MLVA, multiple-locus variable number of tandem repeats analysis; SPA, *Salmonella enterica* serotype paratyphi A; CMCC, Chinese Medical Culture Collection Center.

The dendogram for the MLVA types distribution demonstrates that 23 MLVA types (MLVA 1-23) were identified in the 215 SPA isolates and were grouped into six distinct cluster groups A, B, C, D, E and F. All of the Yuxi isolates were exclusively grouped into cluster C with nine MLVA genotypes (MLVA 10-18; Fig. 2). The 20 CMCC isolates were grouped in cluster A B, D, E and F with 14 MLVA types (MLVA 1-9 and MLVA 19-23; Fig. 2). There are two key observations to note among the 20 CMCC strains. Firstly, the same MLVA type of SPA emerged in different countries. For example, 50501, 50502 and 50506 (Dalian, China) and 50001 (Denmark) were typed as MLVA7, ATCC9150, 50002 (Denmark) and 50701 (Czech Republic) were typed as MLVA19, and 50507 (Lanzhou, China) and 50101 (Former Soviet Union) were typed as MLVA6, although they were from different countries. Secondly, there existed different MLVA types in the same region or country. For instance, one strain from Dalian, China (50505) was identified to be MLVA8, which is different from the other three Dalian strains of MLVA7 (50501, 50502 and 50506). Despite the fact that 50672 and 50674 were from Poland their MLVA types were MLVA9 and MLVA23, respectively. The same phenomena were observed in two strains from Bulgaria 50433 (MLVA2) and 50434 (MLVA5).

**Figure 3:**
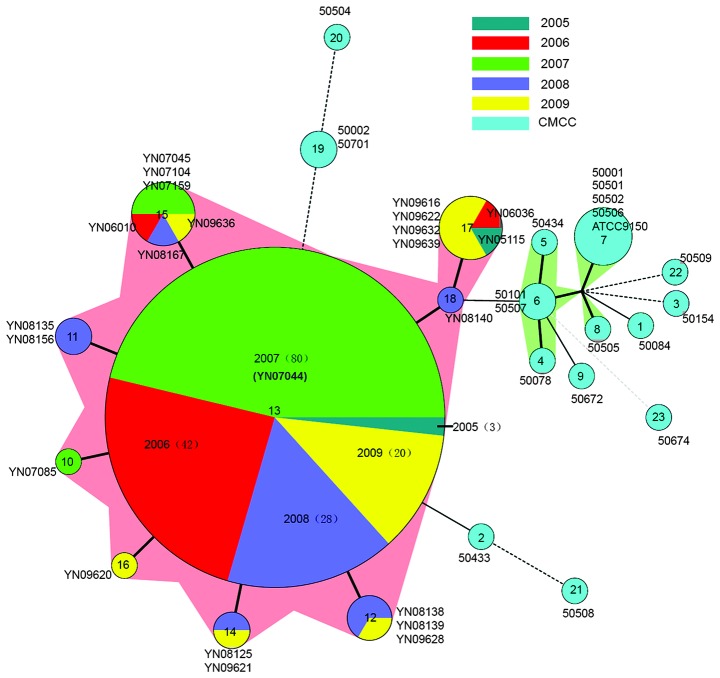
MST obtained from 215 SPA isolates with 23 MLVA types distinguished by nine VNTRs. Each circle represents a particular MLVA type with the same MLVA profiles. The size of the circle reflects the number of isolates whereas the distance between the circles represents the genetic divergence. The heavy short lines connect single-locus variants, the thin long lines connect double-locus variant and the dotted lines connect those MLVA genotypes with differences in >2 loci. The colors represent the years of the isolates or origin of isolates. The halos surrounding the various types denote the grouping obtained by BioNumerics analysis. MST, minimum spanning trees; SPA, *Salmonella enterica* serotype paratyphi A.

The genetic correlations among the 215 isolates were determined based on the MLVA profiles using the MST algorithm (10,23,28). As demonstrated in Fig. 3, MST offers a more detailed view of the diversity of the 215 isolates and highlights the closer subtypes that differ by few allelic changes (23). Isolates with the same MLVA profiles were clustered in a circle. A total of 173 Yuxi isolates form the MLVA13 circle surrounded by 22 other isolates with 8 MLVA types. It indicates that the 195 Yuxi isolates are closely related with each other. Although the 195 Yuxi isolates are distinct from the 20 CMCC strains, they are relatively close to 50433 (Bulgaria) with MLVA2, and also close to 50002 (Denmark), and 50701 (Czech) with MLVA19. YN08140 (Yuxi) with MLVA18 is closely related to 50101 (Former Soviet Union) and 50507 (Lanzhou, China) with MLVA6, which varied in only two VNTR loci (Fig. 2).

### Epidemiology of SPA in Yuxi

Although 195 Yuxi SPA isolates distribute in nine genotypes (MLVA 10-18), they express only one or two VNTR loci that are different from each other (Fig. 2). The MST demonstrated that they are closely related with each other and separated from the 20 CMCC strains (Fig. 3). Table III reveals the MLVA type distribution of 195 Yuxi isolates collected between 2005 and 2009. In all, MLVA13 accounted for 88.7% (173/195) of the Yuxi isolates. Among the 48 outbreak isolates in 2007, MLVA13 accounted for 91.7% (44/48). Outside of the 2007 outbreak, MLVA13 accounted for 87.8% (129/147) of Yuxi sporadic isolates.

## Discussion

A reliable method for subtyping bacterial isolates is a prerequisite for the identification of sources and transmission routes of an infectious disease (29). There is no doubt that PFGE is currently the gold-standard technique for subtyping numerous bacteria, including *Salmonella* serotypes with reproducible patterns and high resolution and is widely used by the CDC PulseNet surveillance program worldwide (30). However, the widespread use of PFGE is limited in the CDC of numerous Chinese cities and counties by the lack of specifically trained personnel, sophisticated and expensive equipment and precise standard protocols (31). Conversely, MLVA, which is based on the evaluation of differences in the number of TRs, is a quick, cheap and simple method for the molecular typing of bacteria (20). In the present study, a MLVA with nine VNTR markers was developed, which exhibited a wide range of variability for subtyping 215 SPA isolates into 23 MLVA types. The phylogenetic association among the 20 CMCC SPA strains with various backgrounds was elucidated clearly with 14 MLVA types. Clonal groups among the 195 Yuxi isolates in the different years were discerned with nine other MLVA types. The outbreak-related isolate was identified to be MLVA13 in 2007. Eight novel SPA isolates separated from patients in 2010 were examined with the MLVA method developed in the present study, and it was identified that six of the isolates were MLVA13 while one was MLVA14 and another was MLVA16. These results indicate that the VNTR markers identified in the present study are applicable to subtype SPA.

Yuxi, a medium-sized city (15,285 km^2^) with 2,095,532 residents distributed into two districts (Hongta and Eshan) and six counties (Chengjiang, Tonghai, Jiangchuan, Huaning, Xinping and Yuanjiang) has been one of the most severely endemic areas of paratyphoid fever in China since 1999. There was a progressive increase in the number of SPA cases in Yuxi between 2005 and 2009. The results of MLVA typing for Yuxi isolates indicate that the MLVA13 isolate was the epidemic clone in Yuxi in outbreaks and sporadic cases. Consistent with the sources and transmission routes of enteric fever (32,33), contaminated water and food are major sources of SPA in Yuxi. It was identified that contaminated well water in a vegetable market of Hongta was the direct factor leading to the 2007 outbreak of SPA. More than 90% of patients in the 2007 outbreak were retrospectively investigated to have purchased vegetables from the Hongta vegetable market near the infected well, where the vendors watered the vegetables using the well water. Subsequently, the SPA isolates were separated from the water in the well. The sources of SPA from the well water were further confirmed by the result of MLVA typing for SPA in the present study, demonstrating that MLVA13 SPA were the major clones isolated from the well water, vegetables and patients during the epidemic. In Asia, SPA may also be transmitted by consumption of contaminated foods from street vendors (4). The contaminated foods sold by street vendors may be important vectors of the SPA sporadic isolates in Yuxi as it is highly common in Yuxi to eat at street vendors with poor sanitary conditions. From the patients who had eaten at street vendors, a variety of MLVA types were separated with the majority being the MLVA13 type of SPA. The incidence rate of enteric fever has decreased significantly and remained at a low level following 2010 with the strengthened surveillance of stock sold in the markets and by street vendors.

A total of 20 SPA isolates have been collected from different countries and regions during different periods by the CMCC thus far. To investigate the Yuxi SPA isolates, the MLVA type of 20 CMCC SPA strains was analyzed, and revealed a large diversity with 14 MLVA types which are unrelated to the 195 Yuxi isolates.

## Figures and Tables

**Table I:** Information of 20 SPA strains collected by CMCC.

Strain	Source
ATCC9150	ATCC
50001	Denmark
50002	Denmark
50084	USA
50101	Former Soviet Union
50154	France
50433	Bulgaria
50434	Bulgaria
50672	Poland
50674	Poland
50701	Czech Republic
50078	Beijing, China
50501	Dalian, China
50502	Dalian, China
50504	Dalian, China
50505	Dalian, China
50506	Dalian, China
50507	Lanzhou, China
50508	Guangdong, China
50509	Guangdong, China

CMCC, Chinese Medical Culture Collection Center; SPA, *Salmonella enterica* serotype paratyphi A; ATCC, American Type Culture Collection.

**Table II:** Characteristics of 9 VNTR loci for 20 CMCC SPA strains and AKU_12601.

VNTR locus	Primer sequence (5′-3′)	Repeat model	Repeat number
TR27	F: GGAAAGACTGGCGAACAAAT		
	R: TCGCCAATACCATGAGTACG	TACTGG	9–16
TR51	F: CCATGGCTGCAGTTAATTTCT		
	R: TGATACGCTTTTGACGTTGC	ACCATG	1–11
TR41	F: TGGGAAACTTATCTTCGA		
	R: TAATCAGTCTGGCCTGTG	ACATCTCCT	1–6
TR43	F: TACTGCTTTCGCCATCGG		
	R: ATAATCCGGGTAAAGACC	CCGTTAACCG	3–5
TR5	F: GCATACACCGCAGCACTC		
	R: TTCCTTTCCCTGCTTATTTGTC	TAGCAGGTAA	2–4
TR40	F: CGGGTGATTCTGTTATCT		
	R: ATAGTGTTACGCACCTCA	TTTTTTAAG	3–6
TR44	F: CAGAAGCAGTTCCACCACCT		
	R: CATTTCACATCGCCGACTTT	GCAGGAGCTGGTGGGCGA	1–3
TR24	F: GCTGAAGAAGCGGCAAAAC		
	R: GTACCGCTATCTTTCGATGGC	45bp^a^	7–8
TR49	F: GCTTGCAGCTAAATGGAT		
	R: ATCTGACGAAAGCGGAAC	232bp^b^	2–3

a Model of repeats: TCGGCAGCCGCTTTCTTCTTAGCGTCCGCCGCTGCTTTCGCCGCC;

b Model of repeats: TTCCC GCTC CAAAATTTGAAAGTACTTGTTAAGTACAGACCACCAATCGCAGGATTTCGAATTGCGACAA GGCGGCAACTGAATGAGTCCT CAGGAGCTTACTGAAGTAAGTGACTGAGGCGAGTGAAGGCAGCCAACGCAGTAGCGGTTCGAAAGACGAAGATTATGC GGGAATAGCTC AGTTGGTAGAGCACGACCTTGCCAAGGTCGGGGTCGCGAGTTCGAGTCTCGT.

CMCC, Chinese Medical culture Collection Center; SPA, *Salmonella enterica* serotype paratyphi A; VNTR, variable number of tandem repeats.

**Table III:** The MLVA types distribution of 195 SPA Yuxi isolates between 2005 and 2009.

	MLVA type									
	
Year	10	11	12	13	14	15	16	17	18	Total
2005				3				1		4
2006				42		1		1		44
2007	1			80		3				84
2008		2	2	28	1	1			1	35
2009			1	20	1	1	1	4		28
Total	1	2	3	173	2	6	1	6	1	195

MLVA, multiple-locus variable number of tandem repeats analysis; SPA, *Salmonella enterica serotype paratyphi A*.
